# Supplementary material for: Enhanced hyperspectral tomography for bioimaging by spatiospectral reconstruction
Source: Sci Rep. 2021 Oct 21;11:20818. doi: 10.1038/s41598-021-00146-4 (PMC8531290; doi:10.1038/s41598-021-00146-4)
Supplement: Supplementary file 1 — Supplementary Information 1. [file 41598_2021_146_MOESM1_ESM.pdf]

Supplementary Information:

Enhanced hyperspectral tomography for bioimaging by spatiospectral reconstruction

Ryan Warr[1], Evelina Ametova[1,2], Robert J. Cernik[1], Gemma Fardell[3], Stephan Handschuh[4], Jakob S. Jørgensen[5,6], Evangelos Papoutsellis[1,3], Edoardo Pasca[3], and Philip J. Withers[1]

[1] Henry Royce Institute, Department of Materials, The University of Manchester, Manchester, M13 9PL, UK

[2] Laboratory for Applications of Synchrotron Radiation, Karlsruhe Institute of Technology, Kaiserstr. 12, D-76131, Karlsruhe, Germany

[3] Scientific Computing Department, Science Technology Facilities Council, UK Research and Innovation, Rutherford Appleton Laboratory, Chilton, Didcot, OX11 0QX, UK

[4] VetCore Facility for Research, University of Veterinary Medicine Vienna, Vienna, Austria

[5] Department of Applied Mathematics and Computer Science, Technical University of Denmark, Denmark

[6] Department of Mathematics, The University of Manchester, Manchester, M13 9PL, UK

X-ray Source Properties

Figure S1 shows typical incident source spectra measured by a single pixel of the detector, taken from flatfield projections acquired prior to scanning the lizard head and powder phantom samples respectively. For the lizard head flatfield, X-ray fluence rate was calculated to be 2.1×10^6 photons cm^{-2} s^{-1}. For the powder phantom flatfield, the X-ray fluence rate was 6.2×10^6 photons cm^{-2} s^{-1}. Distinct peaks are observed in the 20 – 30 keV range, due to fluorescence of the Cd and Te semiconductor materials within the hyperspectral detector. In addition, different levels of scattering

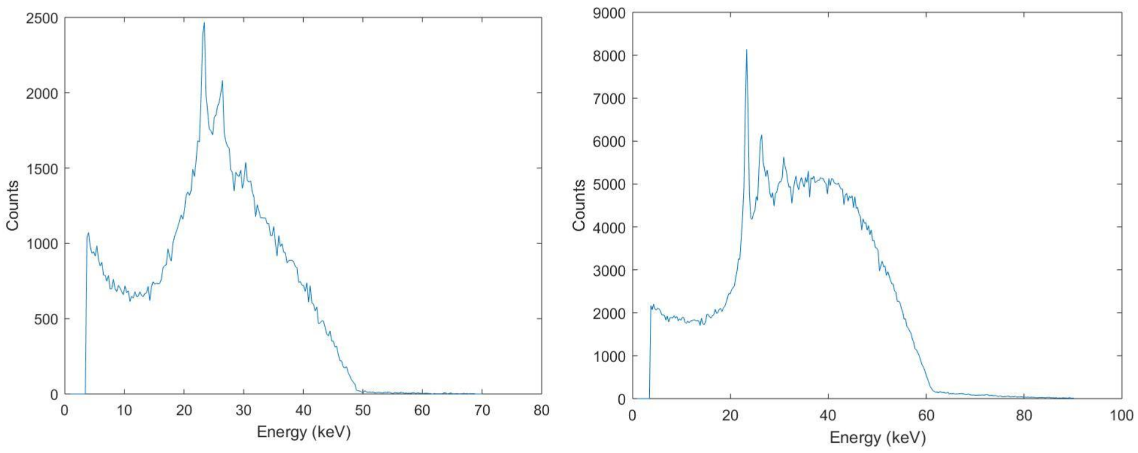

Supplementary Figure S1: Example incident source spectra measured by a single pixel of the hyperspectral detector. For each flatfield, a low-energy tail is observed due to the effect of charge sharing. (Left) Single flatfield projection measured prior to scanning the lizard head sample (50 keV, 0.7 W, and 120 s exposure time.) Sharp peaks appear due to the fluorescence of the Cd and Te materials within the detector. (Right) Single flatfield projection measured prior to scanning the long exposure powder phantom (60 keV, 6W, and 180 s exposure time).

directly affect the proportion of fluorescent events, altering the expected count distribution in the lower energy regions. These changes to the flatfield spectra, compared to those acquired when the samples are in place, directly affect the resulting spectrum in each pixel as flatfield correction is applied. The low-energy tails for each flatfield spectra in Fig. S1 can be attributed to the charge sharing effect.

Spectral Detector Calibration

For both datasets, a calibration was performed using a 'dial source', whereby a ^{241}Am sealed source emits radiation which impinges onto various metal foils. This results in the emittance of X-ray fluorescence photons, which are characteristic of the metals, measured by the detector. Linear fitting of theoretical peak energies to 'channel number' can then be performed, to produce our calibration values. For this study, calibration was performed using the ^{241}Am γ-ray photopeak, as well as the $K_{\alpha,\beta}$ fluorescence peaks of Tb and Ba. Figure S2 (left) shows a typical spectrum measured from a single pixel of the detector, with markers identifying the peaks used for calibration. Calibration values were calculated prior to scanning of each sample, with consistent energy-channel conversions across datasets.

In addition, the calibration data enables the energy resolution of the detector to be measured, by measuring the FWHM of the peaks. Here, we measured the FWHM of the ^{241}Am photopeak (59.5 keV), for every pixel on the detector. Figure S2 (right) shows the distribution of FWHM values, with the energy resolution at 59.5 keV found to be 1.21 ± 0.40 keV. The increased energy resolution, compared to the ideal resolution of 800 eV at 59.5 keV, is attributed to the effect of charge sharing. As seen in Fig. S2 (left), this results in a high number of measured events in the low energy range, as measured charge is distributed between neighbouring pixels, causing a low-energy tail. In addition, the fluorescence of the Cd and Te semiconductor materials can result in additional charge sharing effects.

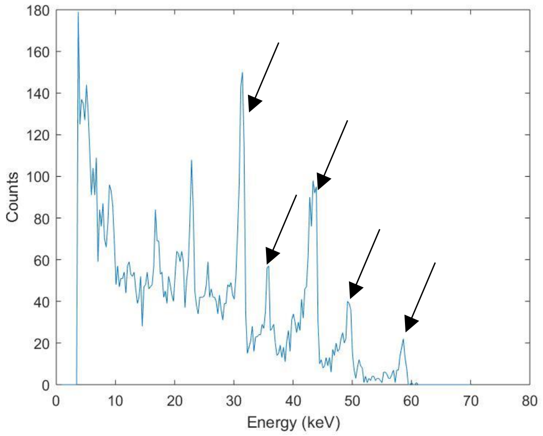

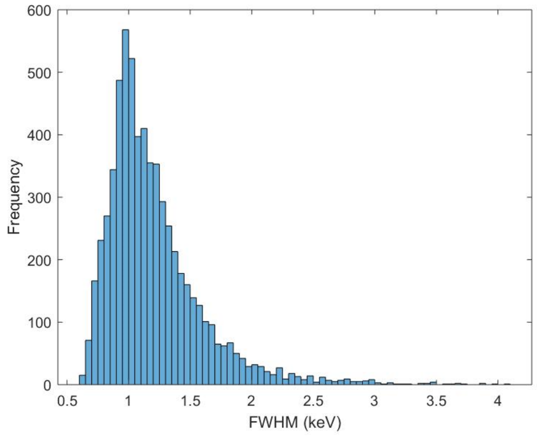

Supplementary Figure S2: (Left) Example spectrum, measured in a single pixel, from the calibration source. Five arrows mark the peaks used for linear fitting, ranging from the $K_{\alpha,\beta}$ fluorescence peaks of Ba (farthest left) to the ^{241}Am γ-ray photopeak (farthest right). A large, low-energy tail is observed due to charge sharing. (Right) Distribution of FWHM values for the ^{241}Am peak, measured at 59.5 keV. Dead pixels were not considered.

Powder Phantom – Phase 1 Artefacts

Following reconstruction of each dataset for the metal phantom, it is observed that phase 1 (the CeO_2 powder), produces slightly higher levels of OD at the edges of the cylinder, with a consistently lower level at the centre extending along the full depth of the cylinder (see Fig. 1b, 2a,b). In conventional white beam CT this 'cupping' contrast is characteristic of beam hardening, but could have a number of causes, for example, uneven filling of the cylinder by the CeO_2 powder.

To investigate this further we scanned a solid tungsten rod and saw the same cupping contrast. This would rule out the effect of uneven packing. Regarding beam hardening, the energy resolution of the system is approximately 1.2 keV, and therefore, while not truly 'monochromatic', the width of each energy channel is very narrow, and it would be expected that the effect of beam hardening would be negligible for any single channel. Nevertheless, the artefact does appear to be much more prominent after the CeO_2 absorption edge than before it (Fig. S3). Consequently, while beam hardening seems unlikely it cannot be unequivocally ruled out. Alternative explanations may lie in the energy detector response and we will continue to investigate this effect.

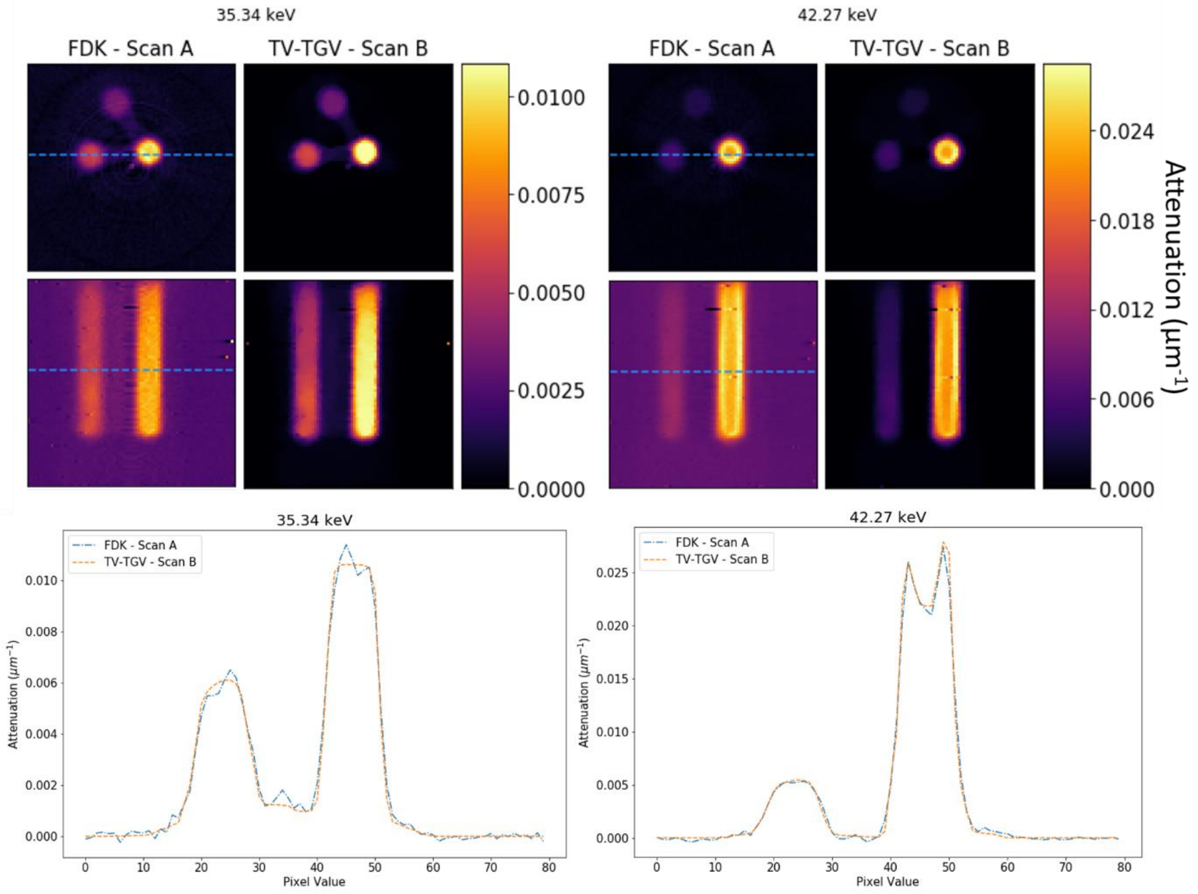

Supplementary Figure S3: (Top) Spatial images of the FDK and TV-TGV reconstructions taken for Scan A and Scan B respectively. Images are shown for single energy channels, both before (top left) and after (top right) the Ce K-edge (40.443 keV). (Bottom) Line profiles taken across two phases, as marked on the spatial images (blue dotted line).

Noise Suppression – Powder Phantom

Given the low noise and clear feature definition of the FDK Scan A reconstruction, we treat this as a ground truth dataset, and as such we may quantify the improvement in reconstruction quality of Scan B by direct comparison with this data. In addition to the CNR values noted in the manuscript, here we also show spatial comparisons of the root-mean-square-error (RMSE) between our Scan B reconstructions and the Scan A dataset. Figure S4 shows RMSE values calculated across two image slices, in the transverse and frontal planes, following FDK and TV-TGV reconstructions of Scan B. RMSE values are shown at a channel just beyond the Ce K-edge, where attenuation is at its highest for this phase, and as such errors are at their highest.

As expected, significant deviations are observed in the RMSE values of Scan B following FDK reconstruction, due to the severity of artefacts and noise present. Such differences are almost entirely removed following TV-TGV reconstruction of Scan B, with only small deviations on the boundaries of the cerium phase, as well as a few erroneously high pixel values.

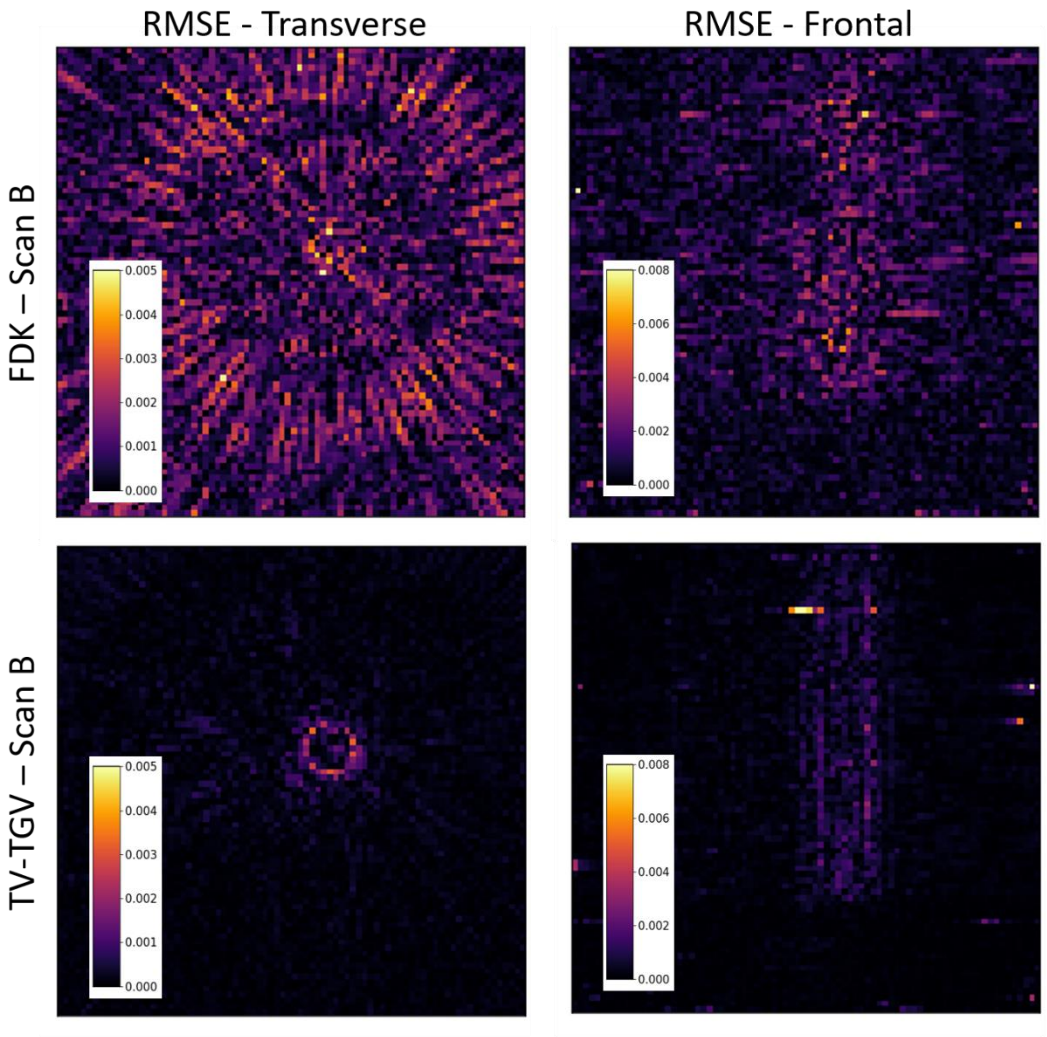

Supplementary Figure S4: RMSE values calculated by comparison of the Scan B reconstructions with the results of Scan A. Images shown are taken for single slices in the transverse (left) and frontal (right) planes, at energy 42.27 keV. Colour bars quantify the RMSE in each image, with scaling consistent within image planes for comparison.

K-edge Subtraction

When the presence of an absorption edge is known, K-edge subtraction (KES) may be used to isolate chemical information due to the element corresponding to the edge. In order for KES to be performed, the energy position of the edge must be known, either through prior knowledge of the sample composition, or visualisation of the acquired spectral profile. Two parameters are then required to determine the data extracted for subtraction either side of the edge: 'Separation' and 'Width', which identify the distance from the edge and the range of energy channels integrated respectively. As illustrated in Fig. S5, the values of each parameter may be adjusted based on the size and shape of the measured absorption step. In the case of the iodine-stained lizard head, optimum values of 5 and 2 channels were found for width and separation respectively.

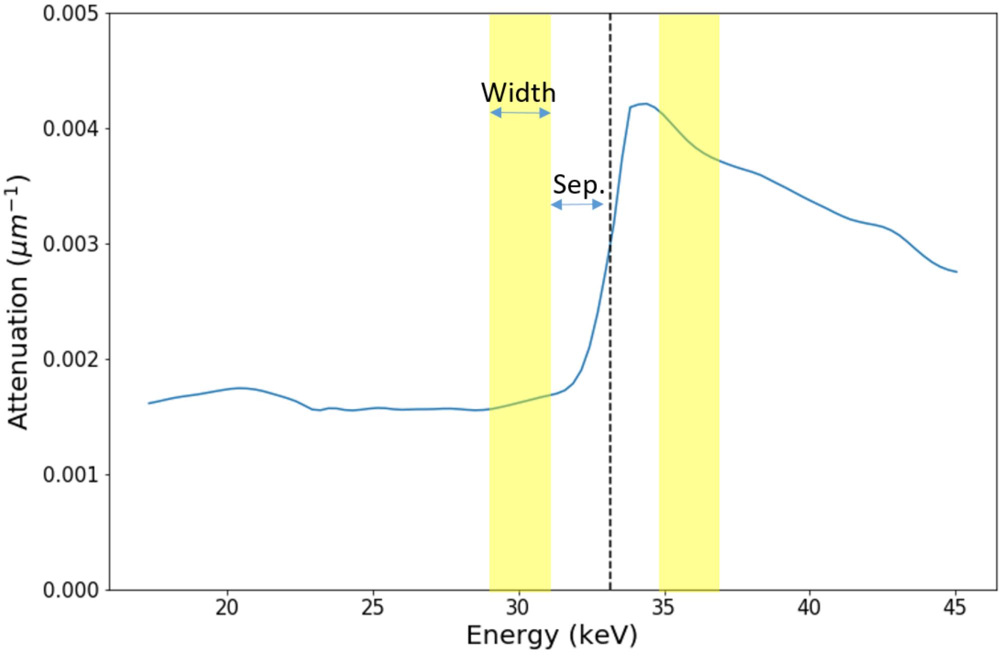

Supplementary Figure S5: Example profile illustrating K-edge subtraction around the iodine absorption edge for a voxel in the stained lizard head. The theoretical position of the edge is marked for comparison (dotted line). The 'separation' (Sep.) determines the distance in energy (channels) away from the edge position, while the 'width' measures the energy (channel) range across which data is extracted (yellow bars). Identical values are used either side of the edge. Sizes of each parameter are not to scale.
